# Atranorin driven by nano materials SPION lead to ferroptosis of gastric cancer stem cells by weakening the mRNA 5-hydroxymethylcytidine modification of the Xc-/GPX4 axis and its expression

**DOI:** 10.7150/ijms.73701

**Published:** 2022-09-25

**Authors:** Zhentian Ni, Xiaoli Nie, Hairong Zhang, Lingquan Wang, Zixiang Geng, Xiling Du, Haiyang Qian, Wentao Liu, Te Liu

**Affiliations:** 1 Department of General Surgery, Ruijin Hospital, Shanghai Jiao Tong University School of Medicine, Shanghai 200025, China.; 2 Shanghai Geriatric Institute of Chinese Medicine, Shanghai University of Traditional Chinese Medicine, Shanghai 200031, China.; 3 Department of Imaging, Dahua Hospital, Xuhui District, Shanghai 200237, China.; 4 Department of Acupuncture, Shanghai General Hospital, Shanghai Jiao Tong University, Shanghai 200086, China.; 5 School of Life Science and Technology, Tongji University, Shanghai 200092, China.

**Keywords:** Atranorin, superparamagnetic iron oxide nanoparticle (SPION), Xc-/GPX4 axis, 5-hydroxymethylcytidine, gastric cancer stem cell (GCSCs), Ferroptosis

## Abstract

Gastric cancer is a highly malignant tumor. Gastric cancer stem cells (GCSCs) are the main causes of drug resistance, metastasis, recurrence, and poor prognosis. As a secondary metabolite of lichen, Atranorin has a variety of biological effects, such as antibacterial, anti-inflammatory, analgesic, and wound healing; however, its killing effect on GCSCs has not been reported. In this study, we constructed Atranorin complexes comprising superparamagnetic iron oxide nanoparticles (SPION) (Atranorin@SPION). *In vitro* and *in vivo* experiments confirmed that Atranorin@SPION could significantly inhibit the proliferation, invasion, angiogenesis, and tumorigenicity of CD44+/ CD24+ GCSCs, and induce oxidative stress injury, Fe_2_+ accumulation, and ferroptosis. Quantitative real-time reverse transcription PCR and western blotting results showed that Atranorin@SPION not only reduced the expression levels of GCSC stem cell markers and cell proliferation and division markers, but also significantly inhibited the expression levels of key molecules in the cystine/glutamate transporter (Xc-)/glutathione peroxidase 4 (GPX4) and Tet methylcytosine dioxygenase (TET) family proteins. The results of high performance liquid chromatography-mass spectrometry and Dot blotting showed that Atranorin@SPION significantly inhibited the mRNA 5‑hydroxymethylcytidine modification of GCSCs. Meanwhile, the results of RNA immunoprecipitation-PCR also indicated that Atranorin@SPIONs significantly reduced the 5-hydroxymethylcytidine modification level of *GPX4* and *SLC7A11* mRNA 3' untranslated region in GCSCs, resulting in a decrease in their stability, shortening their half-lives and reducing translation activity. Therefore, this study revealed that Atranorin@SPIONs induced ferroptosis of GCSCs by weakening the expression of the Xc-/GPX4 axis and the 5-hydroxymethylcytidine modification of mRNAs in the pathway, thereby achieving their therapeutic effect on gastric cancer.

## Introduction

Gastric cancer is a highly malignant digestive tract tumor, which seriously endangers human health 1, 2. A number of studies have confirmed that tumor stem cells are present in gastric cancer tissue, namely gastric cancer stem cells (GCSCs) 3, 4. GCSCs have the characteristics of cancer stem cells, namely high-speed self-proliferation and invasion, drug resistance, and strong tumorigenicity 3-5. GCSCs are proven to be the main cause of metastasis, recurrence, drug resistance, and poor prognosis of gastric cancer 5, 6. Atranorin, a secondary metabolite of lichen, has many biological effects, such as antibacterial, anti-inflammatory, analgesic, and wound healing 7, 8. Recent studies have found that Atranorin not only inhibits lung cancer cell motility and tumorigenesis by affecting activator protein 1 (AP-1), Wnt, and signal transducer and activator of transcription (STAT) signal transduction and inhibiting RhoGTPase activity 9, 10. Atranorin also inhibits breast cancer cell viability *in vitro* by interacting with protein kinase b (Akt) 11. However, whether Atranorin can inhibit the activity of GCSCs is unknown.

Ferroptosis is recently discovered type of programmed cell death that is iron‑dependent and differs from apoptosis, cell necrosis, and autophagy 12-17. The main mechanism of ferroptosis is that under the action of ferrous iron or esterase, unsaturated fatty acids, which are highly expressed on the cell membrane, are catalyzed and lipid peroxidation occurs to induce cell death. This is accompanied by decreased expression of glutathione peroxidase 4 (GPX4) in the antioxidant system (lipid peroxide ) 12-17. When cell are treated with iron ions that remain in the form of ferrous iron, lipid peroxidation could be initiated when iron overload was reached. Liposome peroxide can induce ferroptosis by activating GPX4 in the native digestive system 17. In addition, some studies have found that Ferroptosis suppressor protein 1 (Fsp1) is a glutathione-independent inhibitor of ferroptosis. The Fsp1/CoQ10/NAD (P) H signal transduction pathway exists as an independent parallel system, which together with GPX4 and glutathione, inhibits phospholipid peroxidation and ferroptosis 18. When ferroptosis occurs in cells, the mitochondria become smaller, the membrane density increases, and the number of crista decreases, accompanied by increased lipid peroxidation and increased reactive oxygen species (ROS) in the cytoplasm 19, 20. In addition, the cystine-glutamate antiporter XCT (also known as system Xc- or SLC7A11)/GPX4 axis is important to ferroptosis. Pharmacological inactivation of XC- or GPX4 can induce ferroptosis, suggesting crucial roles for glutathione-dependent antioxidant defenses in preventing ferroptosis 15, 21. High concentration of extracellular glutamate, erastin, or other system Xc- inhibitors block intracellular cystine/cysteine uptake to induce ferroptosis 15, 21. It is important to note that system Xc- function is regulated by glutamate levels, since glutamate is exchanged for cystine in a 1:1 ratio by system Xc-. Accordingly, high extracellular concentrations of glutamate block system Xc- activity, inhibit cystine uptake, and drive ferroptosis 15, 21. Increasing numbers of studies have shown that regulating Ferroptosis is a potential new approach to cancer therapy.

Magnetic nanomaterials generally refer to substances with particle sizes between 0 and 100 nm, which are composed of iron, cobalt, nickel, and their alloys, which can produce magnetism directly or indirectly 22-24. Iron as oxide nanoparticles has been widely studied because of its high magnetic saturation intensity, low toxicity, easy availability of raw materials, and high surface reaction activity 22-24. When the size of magnetic nanoparticles is less than 20 mm, they often exhibit superparamagnetism. Superparamagnetic iron oxide nanoparticles (SPION) have become a research hotspot in gene vectors because of their controllable properties, good stability, and easy modification 22-24. After SPION is combined with plasmid DNA or small interfering RNA (siRNA), nucleic acids can be transfected into mammalian cells under the action of an external magnetic field. The internal and external barriers can be overcome by magnetic adsorption, and the local DNA concentration can be increased to improve the transfection efficiency 22-24. Further studies found that the modification of cationic polymers or cationic liposomes, such as polyethyleneimine, dendrimer, glucose, chitosan on the nanomaterial surface can further realize the interaction between nanomaterials and the nucleic acids to be transferred, which is helpful to improve the transfection efficiency 22-24. Our previous studies have shown that SPION can efficiently bind microRNAs or siRNAs, mediate their transfection into in cells and nematodes, inhibit the proliferation and invasion of tumor cells, or promote the directional differentiation of stem cells 19, 22-26. At the same time, we and other research groups also found that the distribution of tumor cells containing SPION in nude mice can be easily tracked by nuclear magnetic resonance (NMR) detection 25, 27, 28. In addition, there are many reports that SPION can combine with a variety of chemotherapeutic drugs, greatly improving the penetration, killing, and persistence of chemotherapeutic drugs in tumor cells 29, 30.

Based on the above evidence, we used flow cytometry to isolate CD44+/CD24+ GCSCs from surgical tissue samples of patients with gastric cancer and applied Atranorin@SPION to investigate their effect on ferroptosis of GCSCs *in vitro* and *in vivo*. Meanwhile, from the perspective of mRNA 5-hydroxymethylcytidine (hm5C) modification, we further explored the epigenetic mechanism of Atranorin@SPION‑induced ferroptosis of CD44+/ CD24+ GCSCs.

## Materials and methods

### CD44+/CD24+ GCSCs isolation and culture

GCSCs were isolated according to a previously published method 6. In brief, gastric cancer tissues from four patients were first digested using trypsin (containing 0.02% EDTA-Na) at 37 °C for 30 minutes before the reaction was terminated using cell culture medium containing 15% fetal bovine serum (FBS). The volume of the cell suspension was adjusted and 4 μL of fluorescein isothiocyanate (FITC)-labelled rabbit anti-human CD44 monoclonal antibody and Cy3-labelled rabbit anti‐human CD24+ antibody (eBioscience, San Diego, CA, USA) were added to 100 μL of cell suspension and incubated in the dark at 4 °C for 30 minutes. Pre-cooled phosphate-buffered saline (PBS) was used to readjust the volume of the cell suspension to 500 μL. A flow cytometer (BD FACSAria, BD Biosciences, San Jose, CA, USA) was used to select CD44+/CD24+ GCSCs. All cells were resuspended in complete cancer stem cell culture medium: Dulbecco's modified Eagle's medium (DMEM): F12 (HyClone, Logan, UT, USA), supplemented with 10 ng/mL basic fibroblast growth factor, 10 ng/mL epidermal growth factor, 5 μg/mL insulin, 1% bovine serum albumin (BSA), and 5% knockout serum replacement (KnockOut SR) (all from Gibco, Grand Island, NY, USA). The study involving human tissues has been obtained according to consent regulation and approved by the Ethics Review Committee of Shanghai Geriatric Institute of Chinese Medicine of Research in Human Production authorized by Shanghai Municipal Government (No.SHAGE202106998); meanwhile, the informed consent has been provided by all patients conducted in accordance with the Declaration of Helsinki.

### Preparation of SPION bound to Atranorin (Atranorin@SPION)

The Atranorin@SPION was purchased from NOVOBIO (NOVOBIO Biotechnology Co., Ltd., Shanghai, China). In brief, 100 mg of Atranorin in 1.0 ml phosphate buffer (0.2 mol/L Na_2_HPO_4_, 0.2 mol/L NaH_2_PO_4_, pH 6.0) and 100 mg of SPION were mixed and ultrasonicated for 60 min. To isolate the product from the mixture, a neodymium magnet was used. Finally, the product was washed with water and ethanol three times and vacuumed dried at room temperature.

### MTT assay

Briefly, 2000 cells/mL of each group were seeded in the wells of a 96-well plate. After 24 h, 10 μL of 3-(4,5-dimethylthiazol-2-yl)-2,5-diphenyltetrazolium-bromide (MTT) solution (Sigma-Aldrich, St. Louis, MO, USA) was added to each group of cells and incubated at 37 °C for 3 h. The medium was discarded, 150 µl of dimethyl sulfoxide (DMSO) (Sigma-Aldrich) was added to each well, and the plate was shaken for 15 s to mix well. The culture plate was placed in a microplate reader to record the absorbance value at 450 nm. The formula for calculating the cell proliferation inhibition rate (%) was (1-OD value of experimental group of cells - blank/OD value of control group of cells - blank) × 100%.

### Annexin V-FITC/PI staining and flow cytometric analysis

Briefly, the experiment was performed according to the instruction manual of the Annexin V-FITC Apoptosis Detection Kit (Beyotime Biotechnology, Jiangsu, China). Adherent cells were digested using trypsin. The cells were washed with PBS once, centrifuged to remove residual body liquid, and gently resuspended in 195 μL of Annexin V-FITC binding solution. Next, 5 μL of Annexin V-FITC was added, and the sample was gently mixed. Finally, 10 μL of propidium iodide (PI) staining solution was added, and the sample was gently mixed and incubated at 20 °C in the dark for 30 min. The cells were then detected using a flow cytometer (Cytomics FC 500, Beckman Coulter, Indianapolis, IN, USA).

### Transwell migration assay

Briefly, 200 μL of serum-free medium containing 2000 cells/mL cells seeded in the upper chamber of the Transwell apparatus at 8.0 μL/well. A total of 600 μL of complete medium containing 10 % FBS was inoculated into the chamber below the Transwell insert. Cells were cultured at 37 °C, in 5% CO_2_ for 48 h. Cells that adhered to the membrane surface were fixed using 4% paraformaldehyde at room temperature for 30 min, stained with Sigma-Aldrich Chemical for 5 min, rinsed with distilled water twice, and the total number of cells was calculated from three non‑overlapping fields under the microscope.

### Effect of Atranorin@SPION on vascular development of zebrafish embryos

According to a previously published method 31, in a 6-well plate, a total of 30, 24-h post-fertilization zebrafish embryos of the fli1a-EGFP/casper strain (Shanghai Research Centre for Model Organisms, Shanghai, China) were added to each well. DMSO (0.1%) was added to the E3 embryonic culture medium (0.58 g/L NaCl, 0.27 g/L KCl, 0.97 g/L CaCl_2_·2H_2_O, 0.16 g/L MgCl_2_·6H_2_O, 1% methylene blue; pH 7.2; all from Sigma-Aldrich; Merck KGaA) in the control group, and IC50 concentration Atranorin@SPION was used in the experimental group. After 8 h of treatment, the medium was replaced with fresh embryonic culture medium, and the phenotypic changes of zebrafish embryos were photographed under a Nikon SMZ 1500 stereomicroscope (Nikon Corporation, Tokyo, Japan; magnification, ×40). The numbers of fully-developed intersegmental blood vessels (ISVs) and dorsal longitudinal anastomotic vessels (DLAVs) in each embryo were counted. The inhibition rate of angiogenesis was calculated using the following formula (24): % inhibition of angiogenesis = [1-(average number of ISVs in the embryoid body in the experimental group/average numbers of ISV and DLAVs in the embryoid body in the control group)] ×100.

### RNA extraction and Quantitative real-time reverse transcription PCR (qRT-PCR)

According to the instructions of the RNAprep pure Tissue Kit (TIANGEN Biotech (Beijing) Co., Ltd, China), about 20 mg of human tissue samples were taken, added with 800 μL of lysis buffer, ground, and homogenized. The supernatant was taken, added with 200 μL of chloroform, mixed, and centrifuged at 4 °C, 13 400 ×* g* for 15 min. Two volumes of anhydrous ethanol were added to the supernatant, mixed, and centrifuged at 4 °C, 13400 × g, for 30 min. The RNA precipitate was resuspended with 500 μL 75 % ethanol and centrifuged again for 5 min. The supernatant was discarded and the pellet was dissolved in 300 μL of diethyl pyrocarbonate (DEPC) water. The OD260:OD280 ratio (generally controlled between 1.8 and 2.0) was detected using 1 μL RNA solution to determine the purity and total concentration of RNA. According to the instructions of miRcute miRNA First-strand cDNA kit (TIANGEN Biotech), total RNA 20 μL of total RNA (100 ng/μL), 25 μL of 2 × miRNA RT Reaction Buffer, 4 μL of 1 × miRNA RT Enzyme Mix, and 6 μL of RNase-Free deionized water were mixed. The following reactions were performed in a PCR instrument, 42 °C for 60 min for the miRNA poly A tail reaction and reverse transcription; subsequently, the enzyme was inactivated at 95 °C for 3 min. Subsequently, according to the steps of miRcute miRNA qPCR Detection (TIANGEN Biotech) instructions, reagents, samples to be tested, and primers were added to the following system: 10 μL of 2 × miRcute Plus miRNA Premix (with SYBR), 1 μL (10 μM) each of 1 × Forward Primer and Reverse Primer, 4 μL of first strand cDNA, and 4 μL of deionized water. The reactions were performed on a real-time fluorescent quantitative PCR instrument: 95 °C for 15 min; 94 °C for 20 sec; 60 °C for 34 sec, and the fluorescence value was read. The 2^-ΔΔCt^ calculation method was used to determine the relative gene expression level, where ΔCt (cycler threshold_ = Ct_genes - Ct_18S rRNA; ΔΔCt = ΔCt_all_groups - ΔCt_control_group. The mRNA expression level was normalized to the expression level of 18S rRNA.

### Western blotting

Briefly, the total proteins of each group were subjected to 12% SDS-PAGE denaturing gel electrophoresis, and transferred to a polyvinylidene fluoride (PVDF) membrane (Millipore, Billerica, MA, USA). After blocking and washing, primary antibodies were incubated with the membranes at 37 °C for 45 min. After washing, the secondary antibodies were incubated with the membranes at 37 °C for 45 min. The membrane was washed four times with Tris-buffered saline-Tween 20 (TBST) at room temperature for 14 min each time. Then, the immunoreactive protein bands on the membraned detected using an ECL kit (Pierce Biotechnology, Rockford, IL, USA).

### Iron (Fe2+) assay

The intracellular ferrous iron level was determined using an iron assay kit (Abcam, Cambridge, MA, USA; #ab83366) according to the manufacturer's instructions 21.

### Lipid peroxidation (LPO) assay

The relative malondialdehyde (MDA) concentration in cell or tumor lysates was assessed using a Lipid Peroxidation (MDA) Assay Kit (Abcam, #ab118970) according to the manufacturer's instructions 21. Briefly MDA in the sample reacts with thiobarbituric acid (TBA) to generate an MDA-TBA adduct. The MDA-TBA adduct can be quantified colorimetrically (OD = 532 nm). C11-BODIPY dye (Thermo Fisher Scientific, Waltham, MA, USA) was used to detect lipid peroxidation in cells. Oxidation of the polyunsaturated butadienyl portion of the dye results in a shift of the fluorescence emission peak from ~590 to ~510 nm.

### Glutathione (GSH/GSSG) assay

The relative glutathione (GSH) concentration in cells was assessed using a GSH/Glutathione disulfide (GSSG) Ratio Detection Assay Kit (Abcam, #ab205811) according to the manufacturer's instructions 21. Briefly, whole cells were diluted to 1:80 for GSH analysis, and serial dilutions of GSH and GSSG stocks were prepared as standards. A one-step fluorimetric reaction of the samples with the respective assay buffer and probes was carried out for 30 min. The yellow product (5-thio-2-nitrobenzoic acid) was detected spectrophotometrically at 412 nm.

### LC-MS Based mRNA modification Detection

The Liquid chromatography-mass spectrometry (LC-MS) based mRNA modification detection was performed by KangChen Bio-tech (Shanghai, China). Briefly, total RNA samples are qualified by agarose gel electrophoresis and quantified using a Nanodrop instrument (Nanodrop Technologies, Wilmington, DE, USA). mRNA was isolated from total RNA using NEBNext Poly(A) mRNA Magnetic Isolation Module (NEB, Ipswich, MA, USA). Then, the mRNA was hydrolyzed to single dephosphorylated nucleosides using an enzyme mix. Pretreated nucleosides solution was deproteinized using Sartorius 10,000-Da molecular wight cutoff (MWCO) spin filters (Sartorius, Göttingen, Germany). LC-MS analysis was performed on Agilent 6460 QQQ mass spectrometer with an Agilent 1260 HPLC system using Multi reaction monitoring (MRM) detection mode (Agilent, Santa Clara, CA, USA). LC-MS data was acquired using the Agilent Qualitative Analysis software. MRM peaks of each modified nucleoside were extracted and normalized to the quantity of mRNA purified.

### Dot blotting

The total RNAs from each group were spotted onto a Hybond-N + membrane. The spotted RNAs were then cross-linked to the membrane using a UV Crosslinker, the membrane was blocked in 5% BSA, and subsequently incubated with rabbit anti‑5‑hydroxymethylcytidine (hm5C) antibodies (Abcam) and horseradish peroxidase (HRP)-conjugated anti-rabbit secondary antibody (Cell Signaling Technology, Danvers, MA, USA), and finally developed using enhanced chemiluminescence reagents and exposed to imaging film 32.

### RNA immunoprecipitation (RIP)-PCR

RIP-PCR experiments were performed using the Magna RIP RNA-Binding Protein Immunoprecipitation Kit (Millipore, Bedford, MA, USA). Briefly, cells from all groups were lysed (500 μL per plate) in a modified cell lysis buffer used for western blotting and immunoprecipitation (IP) (20 mM Tris, pH 7.5, 150 mM NaCl, 1% Triton X-100, 1 mM EDTA, sodium pyrophosphate, β-glycerophosphate, Na_3_VO_4_, and leupeptin) (Beyotime institute of Biotechnology, Zhejiang, China). After lysis, each sample was centrifuged to clear the insoluble debris and then pre-incubated with 20 μg of protein A agarose beads (Beyotime institute of Biotechnology) by rocking for 30 min at 4 °C, followed by centrifugation and transfer to a fresh 1.5 mL tube. The rabbit anti-5-hydroxymethylcytidine (hm5C) antibodies (Abcam; 1:100) were added and incubated for 90 min before the re-addition of 20 μg of protein A agarose beads to capture the immune complexes. The agarose beads were then washed three times with ice-cold homogenization buffer. Then, the co-precipitated RNAs were isolated by resuspending the beads in TRIzol RNA extraction reagent (Thermo Fisher Scientific), and extracted using the RNeasy Mini kit (Qiagen GmbH, Hilden, Germany). Total RNAs were subjected to reverse transcription using a ReverTra Ace-α First Strand cDNA Synthesis kit (Toyobo Life Science, Lausanne, Switzerland.). PCR amplification was performed for 31 cycles as follows: Denaturation at 95 °C for 30 sec, annealing at 65 °C for 30 sec, and extension at 72 °C for 42 sec, using Easy-Load™ PCR Master Mix (Beyotime Institute of Biotechnology). The amplification products were visualized using 1.2% agarose gel electrophoresis 31, 32.

### *In vivo* xenograft experiments

NOD-scid mice (NOD.CB17-*Prkdc*^scid^/NcrCrl) aged 6-7 weeks and weighing 20-22 g were used in the experiment. The animal study was performed at the Shanghai University of Traditional Chinese Medicine with approval from the Institutional Animal Care and Use Committee in accordance with the institutional guidelines. All mice were randomly divided into two groups, and each group consisted of four mice. In experimental group, approximately 1 × 10^5^ GCSCs in logarithmic growth phase were harvested and inoculated subcutaneously into NOD-scid mice, and intraperitoneal injection of 100 μl Atranorin@SPION (10 mg/kg) every 2 days. In control group, approximately 1 × 10^5^ GCSCs in logarithmic growth phase were harvested and inoculated subcutaneously into NOD-scid mice, and intraperitoneal injection of 100 μl SPION (10 mg/kg) alone every 2 days. After 2 months, the mice were sacrificed, and their tumors were excised. The tumor weight was measured, and the tumor volume was calculated according to the formula: Tumor volume (mm^3^) = (wh^2^)/2, where w is the longest axis (mm) and h is the shortest axis (mm).

### Magnetic Resonance Imaging

According to previously published methods 25, 33, briefly, mice in each group were anesthetized with 1% pentobarbital sodium and placed on an UMR560 1.5 TMRI instrument. The scanning parameters were all fast spin-echo sequences (FSE). T1WI fat suppression, TR488 ms, Te14 ms, layer thickness 5.0 mm, field of view (FOV) 220 mm × 220 mm; t2WI fat suppression, TR 2160 ms, Te 96 ms, slice thickness 5.0 mm.

### Hematoxylin and eosin (H&E) staining

Briefly, tissue samples were fixed with 4% paraformaldehyde, dehydrated, and embedded in paraffin. Sections were cut at 4 μm thickness and placed on a slide. Subsequently, xylene was used for dewaxing, followed by ethanol gradient dehydration. Hematoxylin staining solution was added at room temperature and incubated for 5 min, 1% hydrochloric acid ethanol was used for differentiation for 30 sec, ammonia water was added for 1 min to turn the dye blue, and the slides were washed using distilled water for 5 min. Subsequently, the eosin staining solution was applied at room temperature for 2 min, followed by washing with distilled water for 2 min. The slides were subjected to gradient ethanol decolorization and xylene penetration for 2 min. Finally, the slides were coverslipped and seal with neutral gum.

### Immunofluorescence staining

All fresh tissues were immersed in 4% paraformaldehyde (Sigma-Aldrich) at room temperature for 30 min. Ethanol gradient dehydration, paraffin embedding, slicing (thickness = 6 μm ), and foaming in xylene dewaxing were carried out. The tissue sections were blocked with immunohistochemical blocking solution (Beyotime Biotechnology Co., Ltd.) at 37 °C for 30 min. The blocking solution was discarded and the immunohistochemical cleaning solution (Beyotime Biotechnology Co., Ltd.) was used to wash the sections for 5 min at room temperature three times. Then, primary antibodies were added and incubated at 37 °C for 45 minutes. The antibody was discarded, and the immunohistochemical cleaning solution (Beyotime Biotechnology Co., Ltd.) was used to wash the sections for 5 min at room temperature three times. Then, the secondary antibody was added and incubated at 37 °C for 45 min. The antibody was discarded, and the immunohistochemical cleaning solution (Beyotime Biotechnology Co., Ltd., Zhejiang, China) was used to wash the sections for 5 min at room temperature three times. Finally, immunofluorescence blocking solution (Sigma-Aldrich) was added.

### Statistical analysis

Each experiment was performed as least three times; data are presented as mean ± the standard error (SE) where applicable. Differences were evaluated using Student's t-tests. P values < 0.05 were considered statistically significant. With respect to the ANOVA and limma options, genes with a |log2FC| cutoff > 1 and q < 0.01 relative to pre-set thresholds were considered to be differently expressed genes (DEGs). We executed log-rank tests for the survival analysis.

## Results

### Atranorin@SPION significantly inhibited the *in vitro* activity of CD44+/CD24+ GCSCs

**Figure 1:**
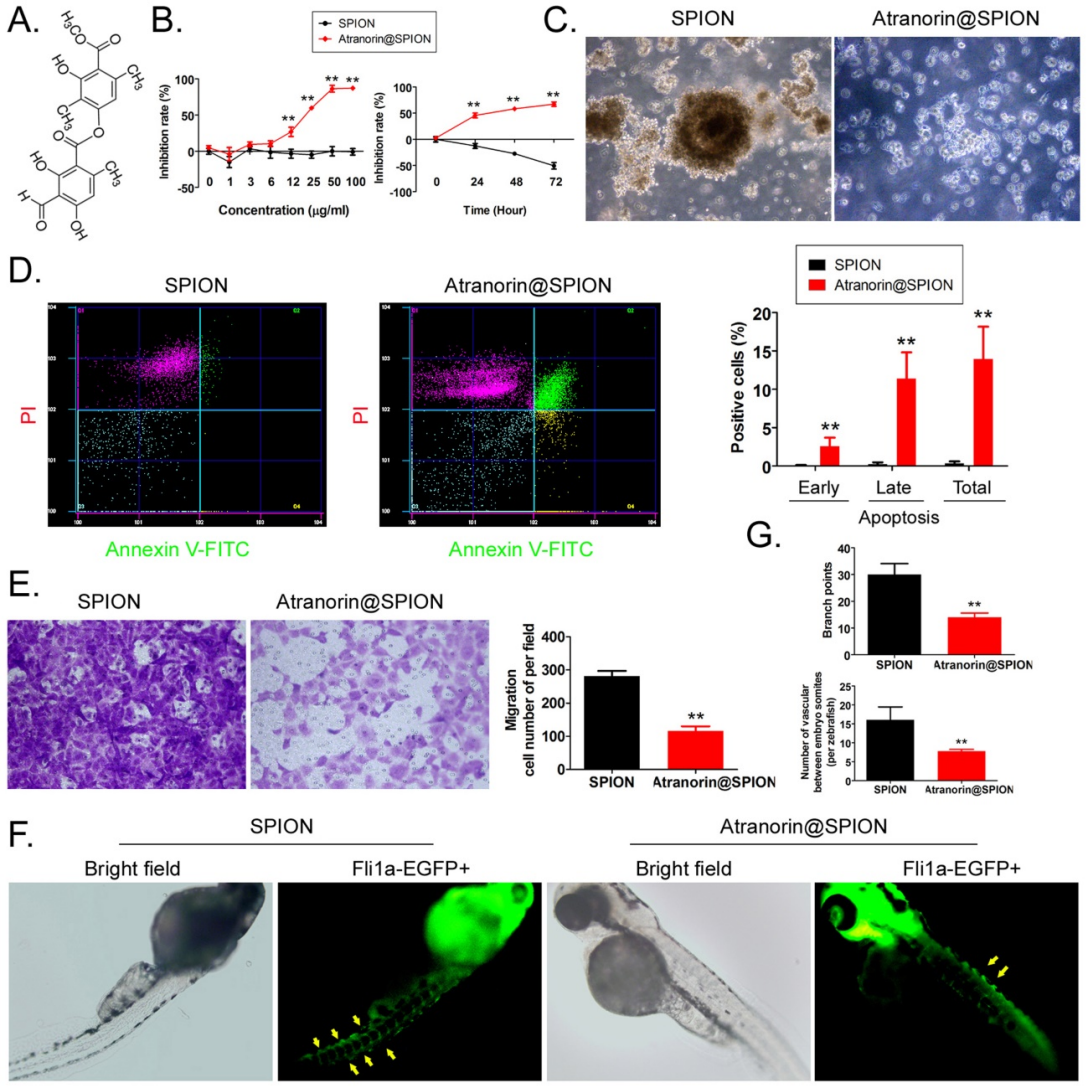
** Atranorin@SPION inhibit the *in vitro* activity of CD44+/CD24+ GCSCs. (A)** Molecular structure of Atranorin. **(B)** MTT results showing that Atranorin@SPION significantly inhibited the proliferation of GCSCs *in vitro*. **p < 0.01 *vs*. SPION; t test; n = 4. **(C)** GCSC phenotype in each group under the light microscope. The magnification was 200 ×. **(D)** The results of flow cytometry showing that Atranorin@SPION significantly induced GCSC apoptosis *in vitro*. **p < 0.01 *vs*. SPION; t test; n = 4. **(E)** Transwell assay showing that Atranorin@SPION significantly inhibited the migration of GCSCs *in vitro*. **p < 0.01 *vs*. SPION; t test; n = 4. **(F)** Atranorin@SPION inhibit the angiogenesis phenotype in Zebrafish. **(G)** Atranorin@SPION inhibit angiogenesis in Zebrafish. **p < 0.01 *vs*. SPION; t test; n = 4.

Atranorin is a secondary metabolite of lichen (Figure 1A). Although it has been reported that Atranorin can inhibit the activity of tumor cells *in vitro* and *in vivo*, its inhibitory effect on tumor stem cells has not been studied. We first loaded different concentrations of Atranorin onto SPION to form Atranorin@SPION complexes. CD44+/ CD24+ GCSCs were treated with the above nano-monomer complexes. The results of the MTT assay showed that when the concentration of Atranorin@SPION was greater than or equal to 12 μg/mL, they could significantly inhibit the proliferation of GCSCs* in vitro* (Figure 1B). The IC50 was 18.977 μg/mL. At the same time, after the GCSCs were treated with Atranorin@SPION complexes at the IC50 concentration, the MTT test results showed that the rate of inhibition of cell proliferation increased significantly over time (Figure 1B, Supplementary data Figure S1). The cell proliferation inhibition rate correlated positively with the Atranorin@SPION concentration and drug treatment time. Moreover, GCSCs in the Atranorin@SPION treatment group were unable to form a clone pellet culture structure (Figure 1C) when cultured *in vitro*. Annexin V/PI staining and flow cytometry showed that the number of normal cells among the GCSCs treated with Atranorin@SPION was much lower than that in the control group at 24 h, while the number of dead cells was significantly higher than that in the SPION control group (Figure 1D). Transwell assays showed that the number of invasive cells in the Atranorin@SPION-treated GCSCs was significantly less than that in the SPION control cells at 24 h (Figure 1E ). In addition, we co-cultured zebrafish embryos with Atranorin@SPION at the IC50 for 24 h. The results showed that the Atranorin@SPION significantly inhibited the growth and development of ISVs and DLAVs in zebrafish, and their rate of inhibition on vascular growth was significantly higher than that of the SPION control group (Figure 1F, 1G). Therefore, the experimental results suggested that Atranorin@SPION significantly inhibited the proliferation and invasion of CD44+/CD24+ GCSCs, and zebrafish angiogenesis, *in vitro*.

### Atranorin@SPION significantly promoted the accumulation of lipid peroxides and the expression of ferroptosis-related genes in CD44+/ CD24+ GCSCs

**Figure 2:**
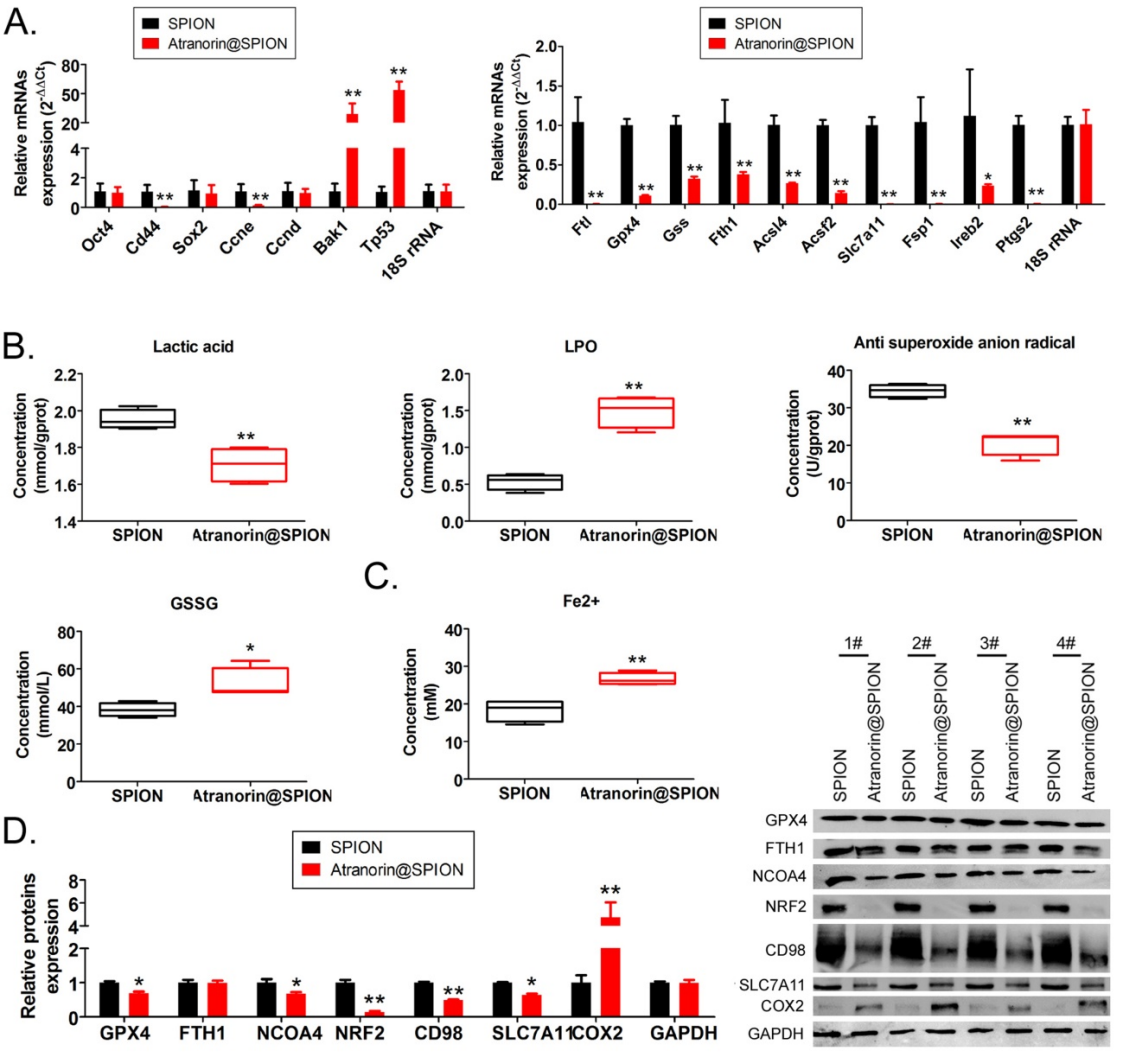
** Atranorin@SPION promoted the accumulation of lipid peroxides and inhibited the expression of ferroptosis-related protective proteins in CD44+/ CD24+ GCSCs. (A)** qRT-PCR results showing that Atranorin@SPION significantly downregulated the mRNA expression of stem cell markers and ferroptosis protective genes in GCSCs. **p < 0.01 *vs*. SPION; *p < 0.05 *vs*. SPION; t test; n = 4. **(B)** The results of biochemical detection showing that Atranorin@SPION significantly downregulated the concentration of lactic acid and anti-superoxide anions in GCSCs, and significantly upregulated extent of LPO and the concentration of Fe_2_+. **p < 0.01 *vs*. SPION; *p < 0.05 *vs*. SPION; t test; n = 4. **(C)** Western blotting results showing that Atranorin@SPION significantly downregulated the levels of ferroptosis-related protective proteins in GCSCs.

The qRT-PCR results showed that Atranorin@SPION significantly inhibited the expression of GCSC stem cell markers *OCT4* and *CD44*, and the gene encoding a cell cycle regulator, *CCNE*; however, the expression of mRNAs encoding apoptosis‑promoting factors (*BAK1* and *TP53*) increased under Atranorin@SPION treatment (Figure 2A). Meanwhile, Atranorin@SPION significantly inhibited the expression of *GPX4*, *FTL*, *SLC7A11*, and *FSP1* in GCSCs (Figure 2B). The western blotting results showed that Atranorin@SPION significantly downregulated the levels of GPX4, NCOA4, BRF2, CD98, and SLC7A11 in GCSCs, and increased the levels of COX2 (Figure 2C). By contrast, the experimental results also showed that the concentration of lactic acid and the extent of LPO in GCSCs in the Atranorin@SPION group were significantly lower than those in the control group, while the concentrations of GSSG and Fe2+ were significantly higher than those in the control group (Figure 2C). Subsequently, the GCSCs were treated on Atranorin@SPION, Atranorin@SPION combined with Ferrostatin-1, Ferrostatin-1 alone, respectively. The results of MTT assay revealed that the cellular growth inhibition rate of Atranorin@SPION combined with Ferrostatin-1 treated group was significantly lower than that of the Atranorin@SPION alone treated group (Supplementary data Figure S2). Thus, above result suggested that Ferrostatin-1 can be weakened cellular proliferation inhibition on Atranorin@SPION via inhibiting ferroptosis. The experimental results suggested that Atranorin@SPION significantly promoted the accumulation of lipid peroxides and the expression of ferroptosis-related genes in CD44+/ CD24+ GCSCs.

### Atranorin@SPION significantly downregulated the mRNA 5‑hydroxymethylcytidine (hm5C) modification level of CD44+/ CD24+ GCSCs

**Figure 3:**
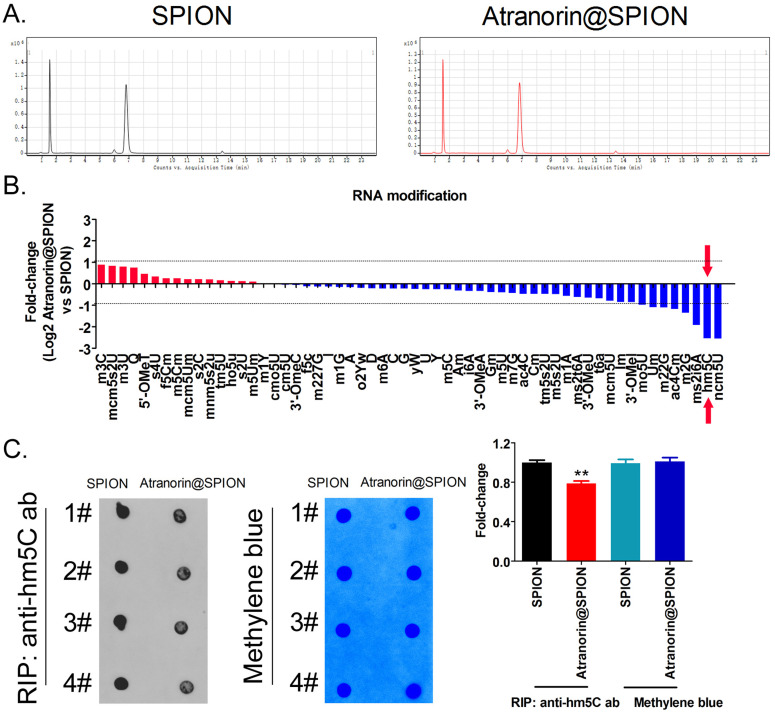
** Atranorin@SPION significantly downregulated the mRNA hm5C modification level of CD44+/CD24 +GCSCs. (A)** HPLC-MS results showing that there were significant differences in multiple mRNA modifications between the two groups. **(B)** Atranorin@SPION significantly downregulated the mRNA levels of hm5C, ncm5U, and m2G in GCSCs. **(C)** Dot blot results showing that Atranorin@SPION significantly downregulated the hm5C modification of GCSCs mRNA. *p < 0.05 *vs*. SPION; t test; n = 4.

**Figure 4:**
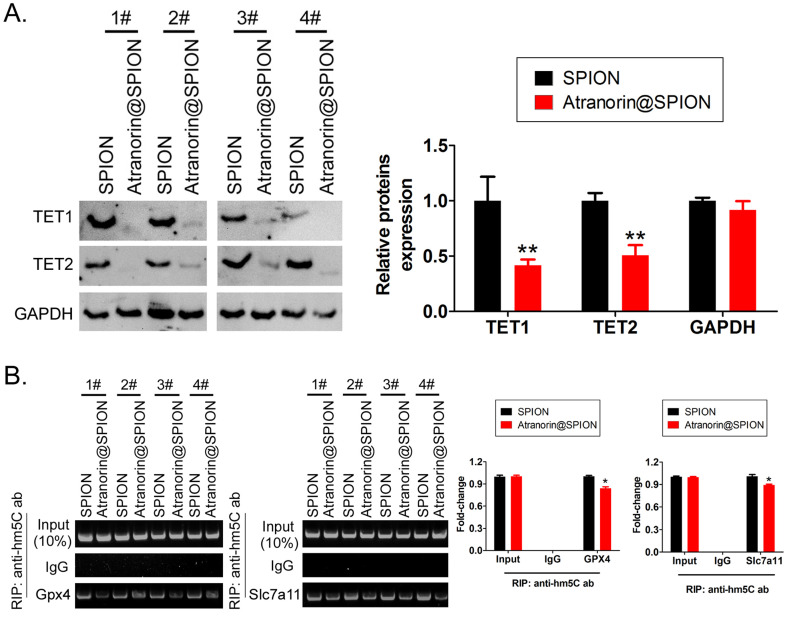
** Atranorin@SPION inhibited the mRNA hm5C modification level of* GPX4* and *SLC7A11.* (A)** Western blotting results showing that Atranorin@SPION significantly downregulated the levels of TET1 and TET2 in GCSCs. **p < 0.01 *vs*. SPION; *p < 0.05 *vs*. SPION; t test; n = 4. **(B)** The results of RIP-PCR indicating that Atranorin@SPION inhibited the mRNA hm5C modification level of *GPX4* and *SLC7A11*. *p < 0.05 *v*s. SPION; t test; n = 4.

The HPLC-MS detection results showed that mRNA hm5C modification levels were significantly decreased in Atranorin@SPION-treated GCSCs, compared with those in in SPION-treated group (Figure 3A, 3B). Meanwhile, dot blotting indicated that the overall mRNA hm5C level in the Atranorin@SPION-treated GCSCs was significantly lower than that in the control group (Figure 3C). Considering that mRNA hm5C modification is regulated by TET family members, we identified the levels of TET family proteins using western blotting. The results showed that the protein levels of TET1 and TET2 in GCSCs treated with Atranorin@SPION were significantly lower than those in the control group (Figure 4A). In addition, the RIP-PCR results showed that a specific product of the *GPX4* and *SLC7A11* mRNAs 3' untranslated regions (UTRs) could be amplified using PCR from each group cells in the complex cross-linked with the anti-hm5C antibody (Figure 4B). However, the amounts of the RIP-PCR amplified products from complexes cross-linked with the anti-hm5C antibody for *GPX4* and *SLC7A11* in the Atranorin@SPION-treated GCSC group were significantly lower than those in the control group (Figure 4B). Therefore, the results suggested that Atranorin@SPION significantly downregulated the hm5C modification level of CD44+/CD24+ GCSC mRNAs.

### Atranorin@SPION attenuated the tumorigenicity of CD44+/ CD24+ GCSCs *in vivo* by inhibiting the expression of GPX4 and SLC7A11

**Figure 5:**
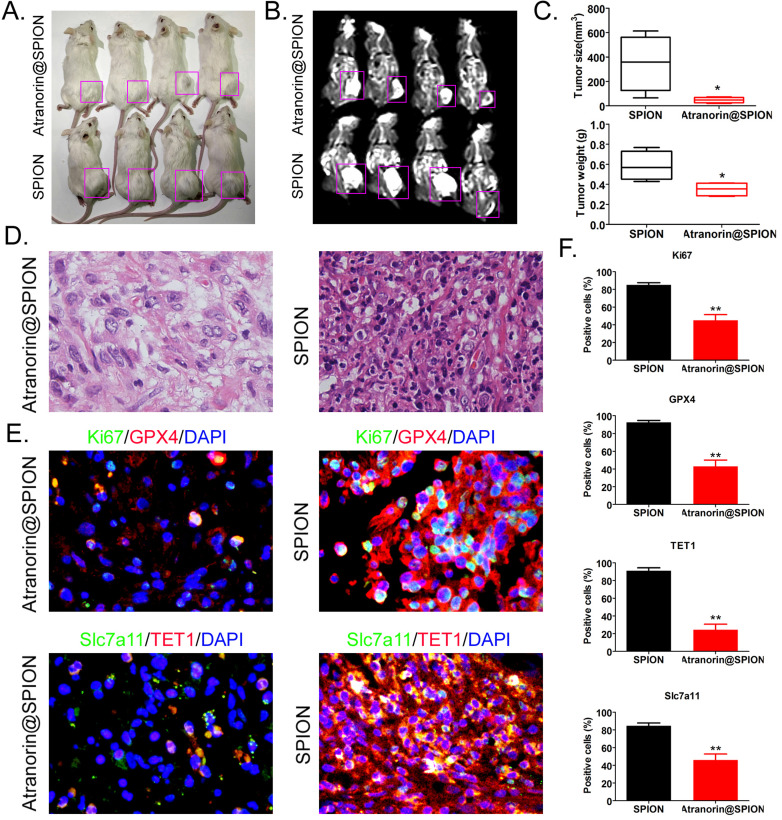
** Atranorin@SPION significantly weakened the tumorigenicity of CD44+/CD24+ GCSCs in immunodeficient mice. (A)** Morphology of dorsal tumors in the tumor‑bearing mice. **(B)** Nuclear magnetic resonance imaging of tumors in the tumor-bearing mice. **(C)** The Atranorin@SPION group had a smaller tumor volume and weight than the control group. *p < 0.05 *vs*. SPION; t test; n = 4. **(D)** H & E staining confirming that the tumor tissues in each group were gastric cancer samples. **(E)** Immunohistochemical staining results showing significantly decreased expression levels of GPX4, SLC7A11, KI67, and TET1 in the Atranorin@SPION group were.

In order to detect the inhibitory effect of Atranorin@SPION treated on tumor activity *in vivo*, we constructed GCSCs tumor bearing mice and continuously intervened with Atranorin@SPION or SPION alone. The body weight of the tumor bearing mice and the size of the tumor on the mice back were measured at the time nodes of 0, 15, 30, 45, 60 day, respectively. The results showed that the tumors on the back of the two groups of mice grew over time (Supplementary data Figure S3A). However, the tumor volumes of Atranorin@SPION treated group mice were significantly smaller than them of SPION alone treated group (Supplementary data Figure S3A). Meanwhile, there was no significant difference in body weight between the two groups (Supplementary data Figure S3B). After 2 months, the naked eye observation showed that the tumors on the backs of the NOD-scid mice injected with Atranorin@SPION were significantly smaller than those in the control group (Figure 5A). The MRI image results showed that the tumor tissues on the backs of NOD-scid mice were high signal intensity on T1WI and obvious high signal intensity on T2WI, indicating the presence of metal substances in the tumor tissues (Figure 5B). After the mice being sacrificed, and their tumors were excised. The tumor tissues from the NOD-scid mice injected with Atranorin@SPION was significantly smaller than that in the control group (Figure 5C), both in weight and volume (Figure 5C). H & E staining showed that although the two groups of tumors were consistent with the pathological characteristics of gastric cancer, multiple cell swelling, nuclear shedding, and nucleoli degeneration were observed in the tumor tissue of the Atranorin@SPION group, and the malignant degree was significantly lower than that of the control group (Figure 5D). The results of immunohistochemical staining showed that the expression levels of KI67, GPX4, SLC7A11, and TET1 in the tumor tissues of NOD-scd mice injected with Atranorin@SPION were significantly lower than those in the control group (Figure 5E). On the other hand, the results of FCM showed that the positive cell ratio of angiogenic biomarkers (CD31 or VEGFR2) in tumor tissue of Atranorin@SPION treated group were significantly lower than those in the control group (Supplementary data Figure S3C). These results suggested that Atranorin@SPION weakened the tumorigenicity of CD44+/CD24+ GCSCs in NOD-scid mice by inhibiting the expression of GPX4 and SLC7A11.

### High expression of *GPX4* and *TET1* suggests poor prognosis in patients with gastric cancer

**Figure 6:**
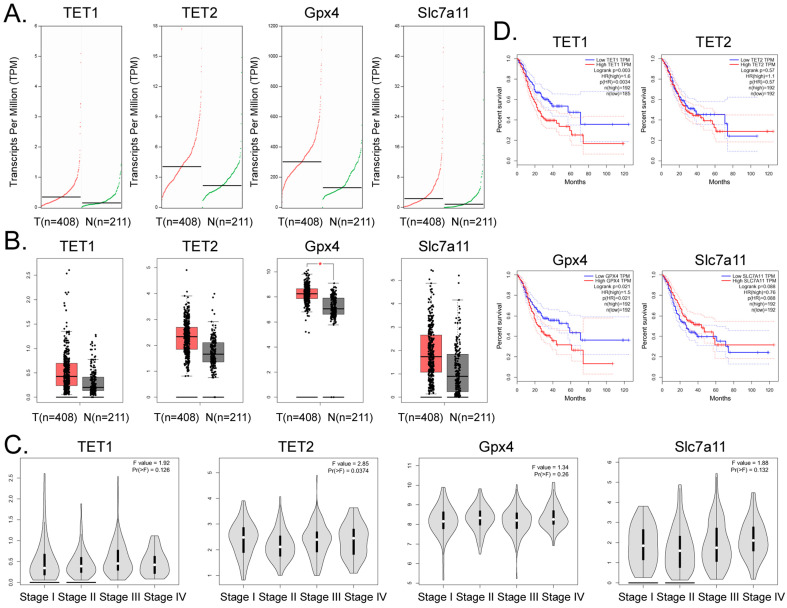
** High expression of *GPX4* and *TET1* suggests poor prognosis in patients with gastric cancer. (A)** Transcriptional copies of *TET1*, *TET2*, *GPX4*, and* SLC7A11* genes in tumor tissues were significantly higher than those in normal tissues. **(B)** The mRNA expression levels of *TET1*, *TET2*, *GPX4*, and* SLC7A11* genes in tumor tissue samples were significantly higher than those in normal control group samples. *p<0.05 vs N. **(C)** There was no statistically significant difference in the expression levels of *TET1*, *TET2*, *GPX4*, and* SLC7A11* genes in gastric adenocarcinoma samples at different stages. **(D)** The statistical results of the survival curve of patients with tumors, indicating that the survival period of patients with gastric cancer with high expression of *TET1* or* GPX4* was significantly lower than that of tumor patients with low expression levels.

Bioinformatic analysis of 408 tumor tissues from patients with gastric adenocarcinoma and 211 tissue samples from non-tumor diseases in the online database GEPIA (http://gepia.cancer-pku.cn/)) showed that the transcript copy numbers of *TET1*, *TET2*, *GPX4*, and *SLC7A11* in tumor tissues were significantly higher than those in the normal tissues (Figure 6A). The mRNA expression levels of these four genes in tumor tissue samples were significantly higher than those in the normal control group, and the expression level of *GPX4* was significantly different (Figure 6B). However, there was no significant statistical difference in the expression levels of the above four genes in each stage of gastric adenocarcinoma (Figure 6C). In addition, the statistical results of the survival curve indicated that the survival period of patients with gastric cancer with high expression levels of *TET1* or *GPX4* was significantly lower than that in patients with low expression of these genes (Figure 6D). Therefore, the clinical data analysis showed that the high expression of *TET1* or *GPX4* correlated positively with an adverse prognosis in gastric cancer.

## Discussion

Nanoparticles are promising drug delivery system and are widely used in preclinical studies of tumor therapy 19, 25, 30, 34, 35. In particular, SPION has been used in tumor tracking, localization, and hyperthermia in experimental animals, because they not only have the basic properties of ordinary nanoparticles, but also have the unique properties of ferrite 28, 30, 33-35. In this study, we combined Atranorin with SPION to form a nano drug delivery system, Atranorin@SPION, which has two distinct advantages. First, SPION can carry a large amount of Atranorin easily into tumor cells to exert its cytotoxic effects and are not easily excluded from tumor cells *in vitro* (our previous studies have confirmed that SPION can efficiently transport non-coding RNAs into cells, but rarely produce drug resistance 19, 22, 25, 26, 33). Second, Atranorin@SPION complexes can easily locate and trace tumor tissues. Our previous studies confirmed that SPION is enriched in tumor cells, and it is easy to detect tumor foci in subcutaneous and visceral tissues of mice using nuclear magnetic resonance 19, 25, 33. In this study, we used Atranorin@SPION complexes to treat mice. We found that as long as the reagents containing SPION was injected, the tumor foci could be located using NMR. This result is very meaningful. It can achieve two purposes of a tumor treatment drug, that is, playing a therapeutic role, and locating and evaluating the size of the tumor by noninvasive imaging detection methods to determine the therapeutic effect of chemotherapy drugs. There have been some reports on the killing effect of Atranorin on tumors 7-11. However, whether Atranorin also inhibits the growth and migration of tumor stem cells has not been reported. Considering that tumor stem cells play a key role in the prognosis and recurrence of cancer, it is of great significance to evaluate the killing effect of Atranorin on tumor stem cells. Interestingly, our results showed that Atranorin could significantly inhibit the *in vitro* and *in vivo* activities of GCSCs. Therefore, the results of this study will provide a good basis for the clinical application of Atranorin.

Next, we explored the mechanism by which Atranorin inhibits GCSCs cell activity. Consequently, we investigated the molecular mechanism of Atranorin@SPION promotion of ferroptosis of GCSCs from the perspective of epigenetic modification. The development of epigenetic transcriptome analysis provides a good platform to reveal RNA epigenetic modifications. To date, more than 170 chemical modifications have been found in RNA 36-41. These modifications are widely distributed in non‑coding RNAs, especially rRNA, tRNA, and snRNA, and are necessary for non‑coding RNAs to exert their normal functions in translation and splicing 36-41. Researchers found that chemical modifications such as N6‑methyladenosine (m6A), N1-methyladenosine (m1A), 5- methylcytidine (m5C), 5‑hydroxylmethylcytidine (hm5C), Inosine, and Pseudouridine (ψ) were also distributed in eukaryotic mRNA, affecting the metabolism and function of mRNAs 36-41. In particular, with the discovery of many mRNA modifying enzymes (Writers, Erasers, and Reader), reversible changes and dynamic regulation of mRNA chemical modification have rekindled researchers' interest 36-41. The most widely studied modifications of mRNA are m6A and m1A 42-44. Many studies have confirmed that m1A and m6A modifications are essential for mRNA stability, splicing, translation, and ncRNA maturation 36-41. The m1A and m6A modifications could stabilize the structure of mRNA, prolong its half-life, weaken the degradation of mRNA by RNAse, and enhance the abundance and translation activity of mRNA. However, some studies have reported that specific m6A modifications reduce the half-life and abundance of mRNA 36-41. Thus, RNA modification is more complex than DNA methylation modification. The methylation modification of cytosine is widely recognized in the DNA field. DNA methylation is usually completed by DNMT1, 3a, 3b and MeCP2. Adding a methyl group to the cytosine 5 covalent bond of CpG dinucleotides in genomic DNA is carried out under the action of DNA methyltransferase 45-47. These modifications will directly lead to a decrease in DNA transcription activity, thereby affecting gene expression. Therefore, DNA methylation plays a negative regulatory role in gene transcription. However, DNA methylation is reversible, usually under the catalysis of the TET family of enzymes (TET1-3). DNA active demethylation is cyclical, starting from 5mC and ending with unmodified cytosine. 5mC is first oxidized to 5hmC, then further oxidized to 5-formylcytosine (5fC), and finally again oxidized to 5-carboxycytosine (5caC) 48-51. Next, thymine DNA glycosylation enzyme (TDG) and base excision repair (BER) jointly removed the 5fC and 5caC from DNA to produce an unmodified cytosine 48-51. Therefore, in DNA, 5 hmC is a demethylation progression from of 5 mC. Once DNA is demethylated, it restores transcriptional and expression activity 48-51. However, in RNA, the process and function of m5C and hm5C are more complex than DNA modification. m5C is widely distributed in tRNA and rRNA. It functions to stabilize the secondary structure of tRNA, influencing the conformation of anti-codon ring and maintaining the fidelity of rRNA translation. The function of m5C is opposite to that of DNA methylation modification 42, 52-55. Recent RNA sequencing results showed that there were more than 8000 m5C sites in the coding and non-coding regions of mRNA, and a considerable number of sites were concentrated in the 5' UTR and 3' UTR regions 42,52-55. The RNA m5C can be catalyzed by methyltransferases NSUN2 or TRDMT1 and oxidized by dioxygenase TET to form hm5C. hm5C may be further oxidized to form f5C and then converted to unmethylated cytosine nucleosides^42^, ^52^-^55^. As a product of m5C oxidation, hm5C can also enhance mRNA translation efficiency, which is similar to the 5hmC modification of DNA 42, 52-55. Studies have shown that hm5C is highly expressed in the brain of Drosophila, hinting at its involvement in brain development 42, 52-55. Thus, m5C and hm5C modifications of RNA are not simple intermediates of methylation and demethylation. Although there have been some reports that the occurrence of ferroptosis is related to RNA methylation modification, the relationship between hm5C modification and ferroptosis has not been reported. We used HPLC-MS to investigate which mRNA modifications changed before and after Atranorin nanocomposite treatment of GCSCs. The results suggested that most mRNA modifications were significantly decreased. Although some modifications such as m3C, m3U, and m5Cm increased slightly, the increase was very small. This result suggested that Atranorin nanocomposites may significantly affect the overall mRNA modification of GCSCs and destroy their mRNA stability. Among the mRNA modifications, the hm5C modification showed the largest decrease. Interestingly, no previous study has reported that Atranorin anti-tumor activities involve RNA epigenetic modification, especially hm5C modification. Subsequently, we detected the hm5C modification of the mRNAs encoding the key proteins GPX4 and SLC7A11 in the ferroptosis regulatory signaling pathway. The results showed that after GCSCs were treated with the Atranorin nanocomposite, the level of hm5C modification of the 3' UTR of the above two mRNAs in cells was significantly reduced. According to existing reports, the mRNA hm5C modification can stabilize its structure and enhance the efficiency of mRNA translation, while the Atranorin nanocomposites significantly reduced the hm5C modification of the 3' UTR of target cell‑specific mRNAs, which undoubtedly reduced the stability and translation efficiency of the mRNA. Without GPX4 and SLC7A11 acting on the metabolism of peroxides and intracellular Fe2+ transport, ferroptosis is prone to occur 13, 56-58.

In summary, this study revealed the molecular biological mechanism by which Atranorin@SPION inhibit the *in vitro* and *in vivo* activity of GCSCs, that is, Atranorin@SPION reduced the expression of members of the Xc-/GPX4 axis and reduced their mRNA 5-hydroxymethylcytidine modification, finally induced ferroptosis of GCSCs.

## Supplementary Material

Supplementary figures.

## Figures and Tables

**Figure 7:**
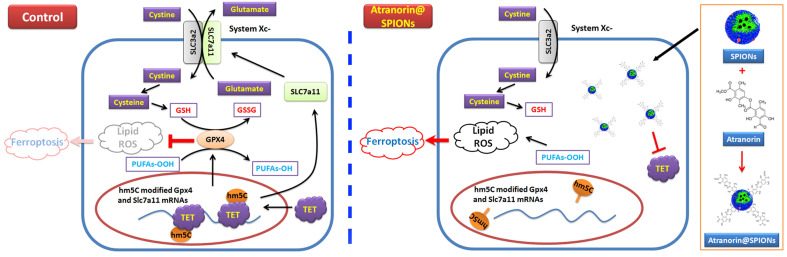
Atranorin@SPION induces ferroptosis in gastric cancer stem cells by weakening the expression of Xc-/GPX4 axis members and their mRNA 5‑hydroxymethylcytidine modification.
