# Supplementary material for: Atranorin driven by nano materials SPION lead to ferroptosis of gastric cancer stem cells by weakening the mRNA 5-hydroxymethylcytidine modification of the Xc-/GPX4 axis and its expression
Source: Int J Med Sci. 2022 Sep 25;19(11):1680–94. doi: 10.7150/ijms.73701 (PMC9553860; doi:10.7150/ijms.73701)
Supplement: Supplementary file 1 — Supplementary figures. [file ijmsv19p1680s1.pdf]

Atranorin driven by nano materials SPION lead to ferroptosis of gastric cancer stem cells by weakening the mRNA 5-hydroxymethylcytidine modification of the Xc-/GPX4 axis and its expression

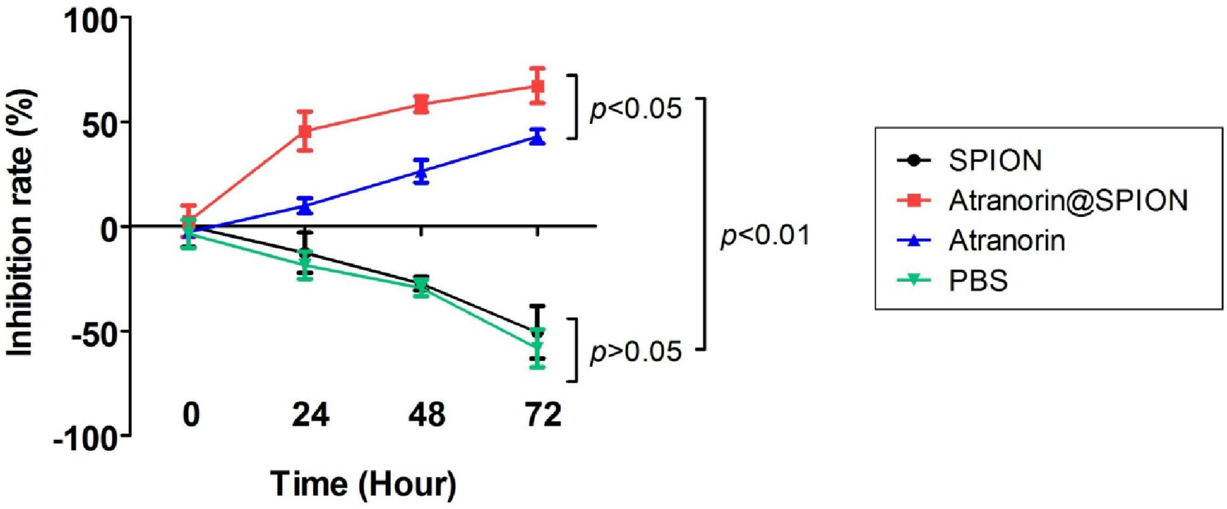

Figure S1 The results of MTT assay

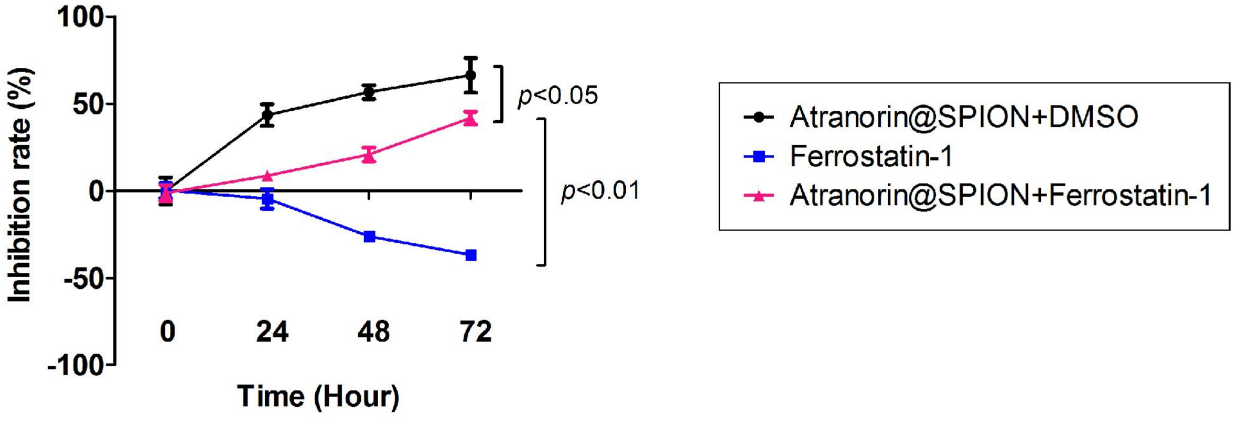

Figure S2 The results of MTT assay

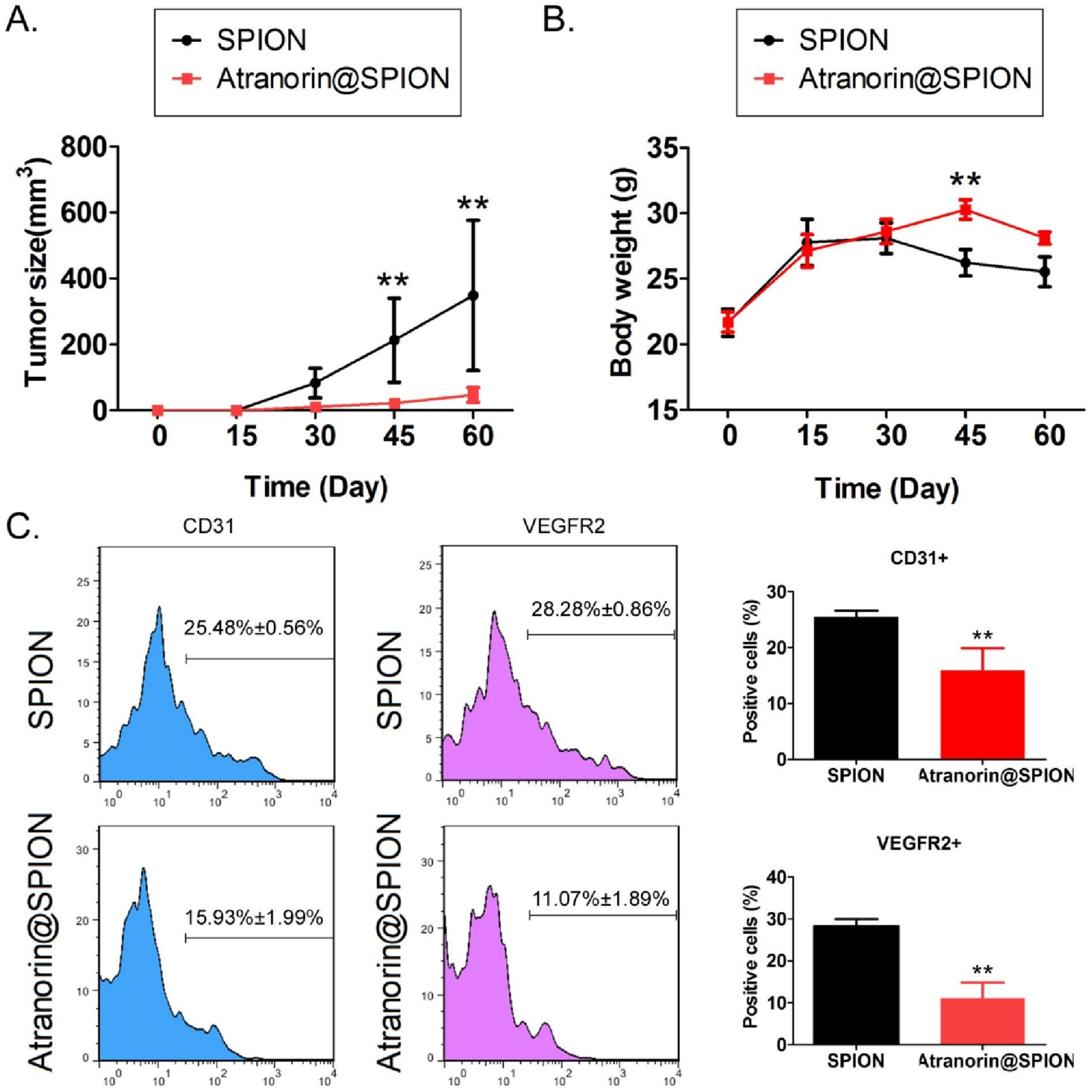

Figure S3 Atranorin@SPION significantly weakened the tumorigenicity of CD44+/ CD24+ GCSCs in immunodeficient mice.

(A) Tumor volume. *p < 0.05 vs. SPION; t test; n = 4. (B) Mice body weight. *p < 0.05 vs. SPION; t test; n = 4. (C) Angiogenic biomarkers (CD31+, VEGFR2) assay. **p < 0.01 vs. SPION; t test; n = 4.
